# E2F4 functions as a tumour suppressor in acute myeloid leukaemia via inhibition of the MAPK signalling pathway by binding to EZH2

**DOI:** 10.1111/jcmm.14853

**Published:** 2020-01-14

**Authors:** Yubin Feng, Lanlan Li, Yan Du, Xiaoqing Peng, Feihu Chen

**Affiliations:** ^1^ The Key Laboratory of Major Autoimmune Diseases of Anhui Province Anhui Institute of Innovative Drugs School of Pharmacy Anhui Medical University Hefei China; ^2^ The Key Laboratory of Anti‐inflammatory and Immune Medicines Ministry of Education Hefei China

**Keywords:** acute myeloid leukaemia, differentiation, E2F4, EZH2, MAPK pathway, proliferation

## Abstract

Acute myeloid leukaemia (AML) is an aggressive and mostly incurable haematological malignancy with frequent relapse after an initial response to standard chemotherapy. Therefore, novel therapies are urgently required to improve AML clinical outcome. Here, we aim to study the dysregulation of a particular transcription factor, E2F4, and its role in the progression of AML. In this study, human clinical data from the Gene Expression Profiling Interactive Analysis (GEPIA) revealed that increased E2F4 expression was associated with poor prognosis in AML patients. Moreover, the experimental results showed that E2F4 was aberrantly overexpressed in human AML patients and cell lines. Depletion of E2F4 inhibited the proliferation, induced the differentiation and suppressed the growth of AML cells in a nude mouse model. By contrast, overexpression of E2F4 promoted the proliferation and inhibited the differentiation of AML cells in vitro. Additionally, E2F4 expression not only is positively correlated with EZH2 but also can bind to EZH2. RNA microarray results also showed that E2F4 can regulate MAPK signalling pathway. EZH2 can reverse the inhibitory effect of E2F4 silencing on MAPK signaling pathway. In summary, our data suggest that E2F4 may be a potential therapeutic target for AML therapy.

## 1 INTRODUCTION

Acute myeloid leukaemia (AML) is characterized by uncontrolled malignant proliferation and impaired apoptosis and differentiation and accounts for 30% of leukaemia‐related paediatric deaths.^1^, ^2^ Although leukaemia research has made great progress in diagnosis, stratification and treatment, this disease is largely incurable, and the overall 5‐year survival rate is still very low at only 25%.^3^, ^4^, ^5^, ^6^, ^7^ Although AML patients have greatly improved after treatment, the prognosis of most patients is still not satisfactory. Chemotherapy and disease recurrence often occur during chemotherapy, which remains a major obstacle to AML treatment.^8^, ^9^

Acute myeloid leukaemia is characterized by high incidence, recurrence and mortality.^10^ Although many effective strategies have been developed to treat AML, such as chemotherapy, supportive therapy and haematopoietic stem cell (HSC) transplantation, the prognosis of this disease remains poor.^8^, ^11^ Therefore, it is important to explore novel avenues for the treatment of AML and to form a better understanding of the molecular mechanisms underlying the treatment of AML.

A large body of literature indicates that the E2F transcription factor family of proteins can regulate cell proliferation. Members of the E2F family contain many important genes that regulate the cell cycle, DNA damage repair and development.^12^, ^13^ E2F4 is a transcription factor (TF) that contributes to controlling the cell cycle. A large number of studies have shown that E2F activity is closely related to cell cycle control.^14^, ^15^ The E2F family of cell cycle regulators is classified as a family of transcriptional activators or inhibitors, but this conclusion has not been well validated.^16^ E2F1‐3‐deficient haematopoietic cells have defects in myeloid cell differentiation, with an accumulation of granulocyte/macrophage progenitor (GMP) cells and a decrease in CD11b^+^ myeloid cells in the bone marrow. Therefore, E2F1‐3 are essential for cell survival and proliferation during the differentiation of bone marrow cells.^17^ However, the role and specific mechanism of E2F4 in AML differentiation and proliferation are still unclear.

In this study, we first studied the expression of E2F4 in human AML patients and cell lines and the association between E2F4 expression and the progression of human AML. We also carried out a series of in vitro and in vivo experiments to knock down E2F4 expression in order to study the effects on proliferation and differentiation. Finally, we used an RNA microarray to detect the gene expression profiles of NB4 cells transfected with E2F4‐targeted short hairpin RNA (shRNA) or negative control shRNA to assess the role of downstream signalling pathways in the carcinogenic function of E2F4.

## 2 MATERIALS AND METHODS

### 2.1 Cell culture

We purchased human normal monocyte cell line SC and NB4 and THP‐1 cell lines from the Shanghai Institute of Cell Biology, Chinese Academy of Sciences; cultured them in Roswell Park Memorial Institute (RPMI) 1640 medium (HyClone) supplemented with 10% foetal bovine serum (FBS); and incubated them in a 5% CO_2_, 37°C environment.

The E2F4 shRNA and negative scrambled shRNA were synthesized by Hanbio (Shanghai, China). NB4 and THP‐1 cells were plated at a density of 1 × 10^5^ cells/well in 24‐well plates for transfection. Then, 30 µl shRNA was added to each well, allowed to stand at room temperature for 15 minutes and placed in a cell culture incubator; the medium was changed after 24 hours.

E2F4 shRNA sense: GATCCGCAGAAGCGGCGGATTTACGACATTATTCAAGAGATAATGTCGTAAATCCGCCGCTTCTGTTTTTTG.
E2F4 shRNA antisense: AATTCAAAAAACAGAAGCGGCGGATTTACGACATTATCTCTTGAATAATGTCGTAAATCCGCCGCTTCTGCG.

### 2.2 Western blot analysis

The cells or tissues were fully lysed with lysis buffer. Proteins were separated using 12% dodecyl sulphate and sodium salt/polyacrylamide gel electrophoresis (SDS/PAGE), and then, the proteins were transferred onto polyvinylidene fluoride (PVDF) membranes (Millipore) and blocked with skim milk for 2 hours before they were incubated with primary antibodies. The membranes were incubated overnight, incubated with secondary antibody for 2 hours at room temperature and visualized using the enhanced chemiluminescence (ECL) system (Thermo Fisher Scientific). The primary antibodies for cyclin A2, E2F4, EZH2, CDK4, ERK, p‐ERK, MAPK, p‐MAPK, cyclin D1, P‐Rb, PCNA, CD11b and CD14 (Abcam) were used at a 1:1000 dilution. The primary antibody for β‐actin (Bioss, Beijing, China) was used at a 1:500 dilution.

### 2.3 RNA extraction and quantitative real‐time PCR (qPCR) analysis

Total RNA was isolated from NB4 and THP‐1 cells with the Total RNA Isolation (TRIzol) reagent (Invitrogen Corp.) and reverse‐transcribed using the First Strand cDNA Synthesis Kit (Thermo Fisher Scientific) to synthesize cDNA. The PikoReal RT‐PCR kit (Thermo Fisher Scientific) was used to measure the relative quantities of mRNA. Expression levels were normalized to the expression of β‐actin.

The primers for targeted genes were as follows:

E2F4: forward 5’‐GACCCCACAGGTGTTTTG‐3’ and reverse 5’‐CCAGGTTGTAGATGTAATCG‐3’; and
β‐actin: forward 5′‐CGCCGCCAGCTCACCATG‐3' and reverse 5'‐CACGATGGAGGGGAAGACGG‐3'.

### 2.4 Differentiation marker analysis

The treated cells were collected and washed twice with PBS, and 1 µL (CD11b‐PE/CY5; CD14‐FITC) antibody was added to each tube. Each tube was placed on ice, gradually cooled on a 100‐r/min shaker and then detected by CytoFLEX (Becton Dickinson, USA). The data were processed using CytoFLEX.

### 2.5 Cell cycle analysis

The treated cells were collected, washed twice with phosphate‐buffered saline (PBS) and then fixed in 70% cold ethanol at −20°C overnight. After centrifugation of the cells at 1000 × g for 5 minutes, the cells were resuspended in PBS for 5 minutes. RNase (20 µL) was added to each tube at 37°C for 30 minutes in the dark, and then, 400 µL PI was added and gradually cooled on ice for 30 minutes (Beyotime). The cell cycle was analysed by a CytoFLEX cytometer (Becton Dickinson). The data were processed using the ModFit software (Verity Software House).

### 2.6 Cell proliferation

The Cell Counting Kit‐8 (CCK‐8) (Bestbio) assay was conducted to analyse cell proliferation. Cells were plated in 96‐well plates at a density of 5000 cells per well and cultured overnight. The number of viable cells was quantified every 24 hours for 3 days. Subsequently, the absorbance of each well was measured at 450 nm using a microplate reader (Thermo Fisher Scientific).

### 2.7 Double immunofluorescent staining

For double immunofluorescent staining, cells were seeded in a six‐well plate and fixed with 4% paraformaldehyde. Afterwards, the cells were blocked with 10% BSA for 10 minutes and incubated with antibodies against E2F4 and EZH2 overnight at 4°C. Then, the cells were incubated with secondary antibody. DAPI was used to counterstain the nuclei, and cells were visualized with a laser scanning confocal microscope.

### 2.8 Tumour xenografts

We purchased 6‐week‐old male NCG nude mice from the Nanjing Model Animal Institute. The mice were allowed to adapt to the SPF environment of Anhui Medical University (Hefei, China) for 1 week. The mice were randomly divided into two groups, and NB4 cells (1 × 10^6^) that were stably transfected with non‐targeting shRNA or E2F4 shRNA were injected subcutaneously. After eight weeks, the mice were killed, and the tumours were removed for weighing. This study was approved by the Ethics and Research Committees of Anhui Medical University; the study was conducted in accordance with the National Institutes of Health Guide for the Care and Use of Laboratory Animals.

### 2.9 Samples

Bone marrow samples were obtained from newly diagnosed AML patients and healthy volunteers who were recruited from Anhui No. 1 Hospital. After bone marrow puncture, a total of 3 to 5 mL of the bone marrow was collected. We used standard Ficoll‐Hypaque density centrifugation to obtain peripheral blood mononuclear cells (PBMCs) from each sample. This study was approved by the Research Ethics Committee of Anhui No. 1 Hospital. Written informed consent was obtained from a parent or legal guardian in accordance with the Helsinki Declaration. All experiments were repeated at least three times.

### 2.10 Co‐immunoprecipitation (Co‐IP) assay

Co‐IP was analysed in NB4, THP‐1 and PBMCs by using the Co‐IP Pull‐Down Kit (Ribobio, Guangzhou). Cells were lysed in a pre‐chilled lysis buffer (with phosphatase inhibitors and protease inhibitors added before use). Protein A beads were incubated with anti‐E2F4 for 4 hours and then incubated with total protein lysates overnight. Western blot analysis of the precipitated protein was conducted as previously described in the Western blot analysis section.

### 2.11 Statistical analysis

All values are presented as the mean ± SD of at least three independent experiments unless otherwise stated. The significant differences between groups were analysed by one‐way analysis of variance (ANOVA) followed by Duncan's test. A *P*‐value <.05 was regarded as statistically significant (*P < .05, **P < .01).

## 3 RESULTS

### 3.1 E2F4 is aberrantly overexpressed in AML and is associated with poor prognosis

**Figure 1:**
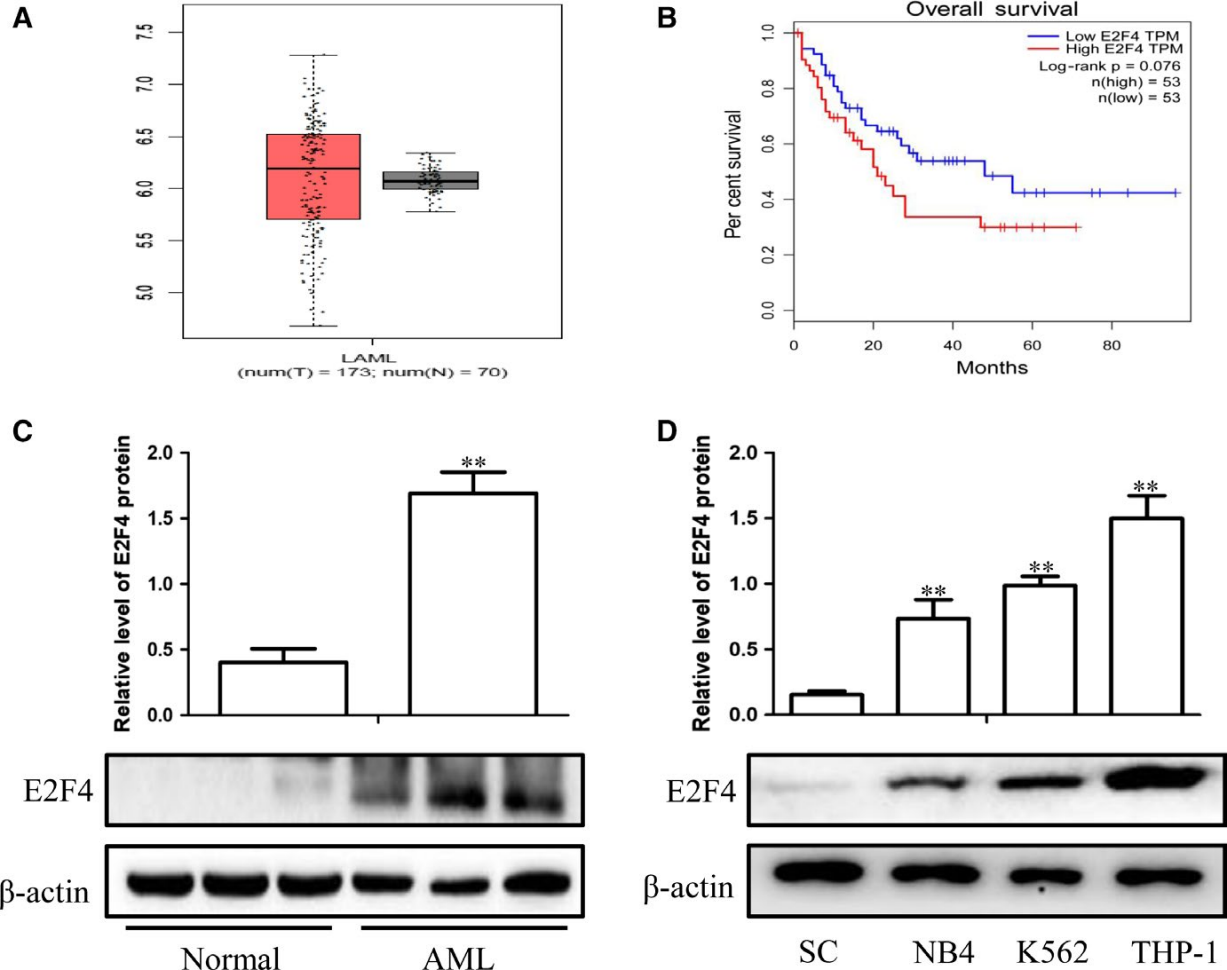
E2F4 is aberrantly overexpressed in AML and is associated with poor prognosis. A, Box plots for E2F4 gene expression in AML and normal tissues from GEPIA. B, Kaplan‐Meier survival plot of overall survival of AML patients in GEPIA, categorized according to E2F4 gene expression (high vs. low, based on mean expression). C, Western blot analysis of E2F4 protein levels in AML and normal tissues. D, Western blot analysis of E2F4 expression in leukaemia cell lines and SC cells. β‐Actin served as a loading control. Data are presented as the mean ± SD of three independent experiments. *P < .05, **P < .01, compared with the normal group

To investigate the role of E2F4 in human AML, we first analysed available human data sets of AML patients in the GEPIA database. We found that mRNA levels of E2F4 were significantly higher in AML tissues (left bar) than in normal tissues (right bar) (Figure 1A) (http://gepia.cancer-pku.cn/detail.php). GEPIA human clinical data revealed that AML patients with higher E2F4 mRNA expression (red bar) exhibited significantly worse overall survival (Figure 1B), suggesting that E2F4 may play a role in promoting AML progression. To confirm the above findings, E2F4 expression was evaluated in the bone marrow of AML patients and the healthy individuals by Western blotting analysis. The assays showed that the protein expression level of E2F4 was significantly higher in the AML patients than in the healthy donors (Figure 1C). In addition, the E2F4 expression was significantly elevated in a number of leukaemia cell lines compared with that in the normal human monocytes (Figure 1D). In summary, these results indicate that E2F4 is abnormally up‐regulated in AML and is associated with poor prognosis.

### 3.2 Lentiviral transduction to knock down or overexpress E2F4 in leukaemia cell lines

**Figure 2:**
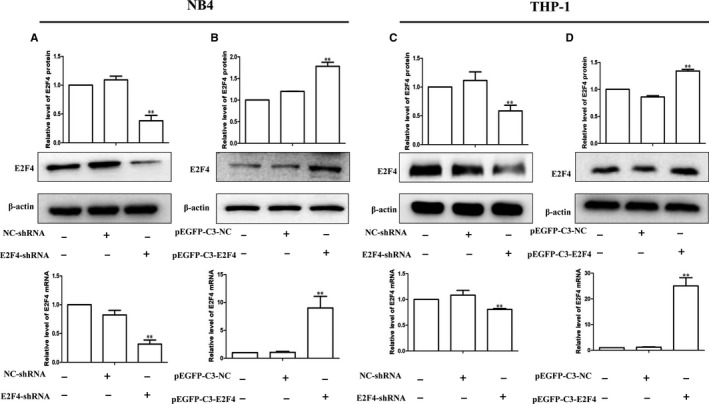
Lentiviral transfection to construct E2F4 knockdown or overexpression in leukaemia cell lines. A, C, Protein and mRNA levels of E2F4 were detected in NB4 and THP‐1 cells without transfection (lane 1) or transfection with negative control (NC; lane 2) or si‐E2F4 (lane 3). B, D, Protein and mRNA levels of E2F4 were detected in NB4 and THP‐1 cells without transfection (lane 1) or transfection with NC (lane 2) or EGFP‐C3‐E2F4 (lane 3). β‐Actin served as a loading control. Data are presented as the mean ± SD of three independent experiments. *P < .05, **P < .01, compared with the NC group

To confirm the function of E2F4 in leukaemia cells, we used lentiviral transfection to silence E2F4 expression in NB4 and THP‐1 cells. The results showed that E2F4‐shRNA reduced E2F4 protein and mRNA levels in NB4 and THP‐1 cells compared to those in the NC group (Figure 2A, 2). In contrast, E2F4 protein and mRNA levels were significantly increased by overexpressing E2F4 via lentivirus in NB4 and THP‐1 cells (Figure 2B, 2).

### 3.3 Effects of E2F4 on the proliferation ability of AML cells in vitro

**Figure 3:**
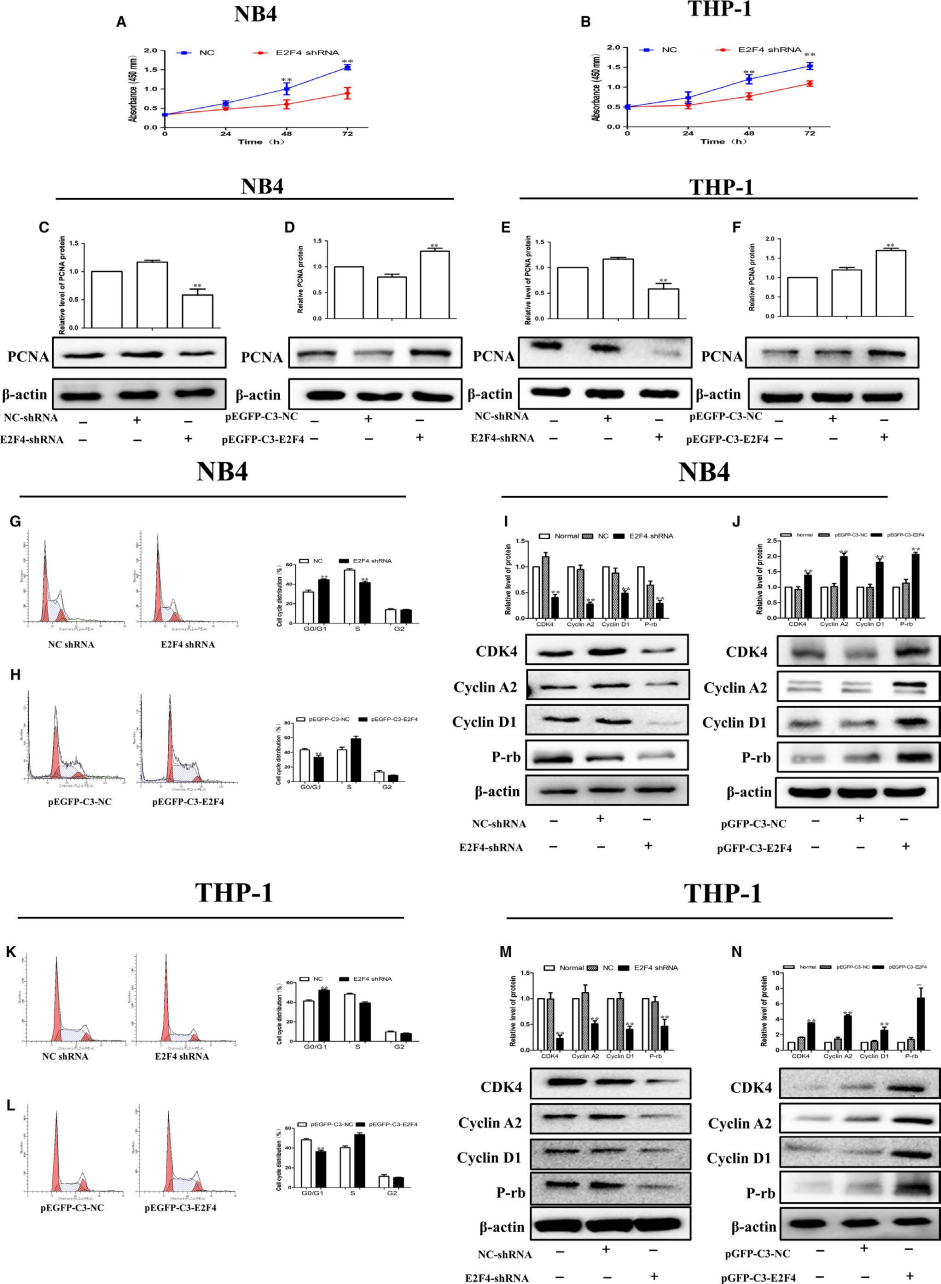
Effects of E2F4 on the proliferation ability of AML cells in vitro. A, B, Cell growth of NB4 and THP‐1 cells after transfection with si‐NLRC5 as determined by the CCK‐8 assay at different time points. C, E, Western blot analysis of PCNA in E2F4‐depleted NB4 and THP‐1 cells. D, F, Western blot analysis of PCNA in NB4 and THP‐1 cells overexpressing E2F4. G, H, Knockdown of E2F4 induced G0/G1 phase arrest in NB4 and THP‐1 cells according to flow cytometry analysis. K, L, Cell arrest in G0/G1 phase was decreased in NB4 and THP‐1 cells transfected with pEGFP‐C3‐E2F4. I, J, Western blot analysis of cyclin D1, cyclin A2, P‐Rb and CDK4 protein levels in NB4 cells after E2F4 depletion or overexpression. M, N, Western blot analysis of cyclin D1, cyclin A2, P‐Rb and CDK4 protein levels in THP‐1 cells after E2F4 depletion or overexpression. β‐Actin was used as the loading control. Data are presented as the mean ± SD of three independent experiments. *P < .05, **P < .01, compared with the NC group

We established stable cell lines with E2F4 knockdown or overexpression. We first performed a CCK‐8 assay to detect the effect of E2F4 on the proliferation of NB4 and THP‐1 cells. The results showed that the E2F4 knockdown group inhibited cell proliferation more than the NC group (Figure 3A, 3). In addition, we also found that the expression of PCNA, a cell proliferation marker, was significantly reduced after silencing E2F4 (Figure 3C, 3). In contrast, the PCNA protein expression levels were increased in E2F4‐overexpressing NB4 and THP‐1 cells compared to those in the control cells (Figure 3D, 3). Next, we used flow cytometry to investigate whether E2F4 knockdown induced leukaemia cell cycle arrest. The flow cytometry results showed that the proportion of G0/G1 population in the E2F4‐depleted leukaemia cells was higher than that in the NC group cells (Figure 3G,K), indicating that E2F4 knockdown blocked cell cycle progression. In contrast, the percentage of G0/G1 phase cells was reduced in E2F4‐overexpressing NB4 and THP‐1 cells compared to that in the control cells (Figure 3H,L). The G0/G1 marker proteins cyclin D1, cyclin A2, P‐Rb and CDK4 also changed. The results showed that the expression of the G0/G1 marker proteins was significantly decreased after silencing E2F4 (Figure 3I, M). In contrast, forced overexpression of E2F4 in NB4 and THP‐1 cells resulted in increased expression of the G0/G1 marker proteins (Figure 3J,N). These results demonstrate that E2F4 expression levels are associated with leukaemia proliferation.

### 3.4 E2F4 mediates the differentiation of leukaemia cells in vitro

**Figure 4:**
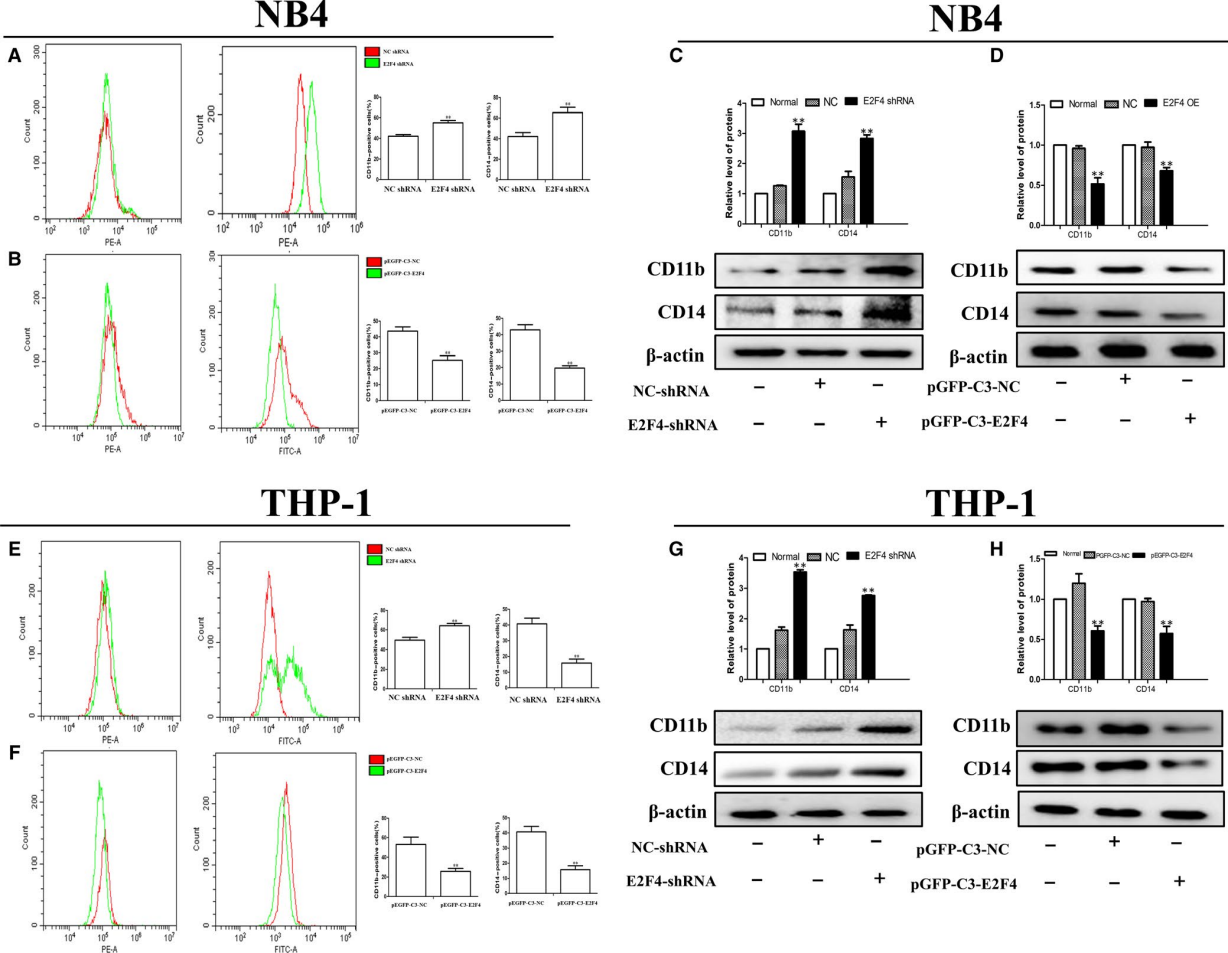
Effects of E2F4 on cell differentiation of AML cells in vitro. A, B, Knockdown of E2F4 induced differentiation in NB4 and THP‐1 cells according to the flow cytometry analysis. E, F, Differentiation markers were decreased in NB4 and THP‐1 cells transfected with pEGFP‐C3‐E2F4. C, D, Western blot analysis of CD11b and CD14 protein levels in NB4 cells after E2F4 depletion or overexpression. G, H, Western blot analysis of CD11b and CD14 protein levels in THP‐1 cells after E2F4 depletion or overexpression. β‐Actin was used as the loading control. Data are presented as the mean ± SD of three independent experiments. *P < .05, **P < .01, compared with the NC group

We previously studied the effects of E2F4 on cell proliferation. It is well known that the pathogenesis of leukaemia is blocked by differentiation. We used a series of experimental methods to detect whether E2F4 could affect leukaemia cell differentiation. The flow cytometry results showed that knockdown of E2F4 promoted the differentiation of NB4 and THP‐1 cells (Figure 4A,4). In contrast, cell differentiation was reduced in the E2F4‐overexpressing NB4 and THP‐1 cells compared to that in the control cells (Figure 4B,4). A previous study showed that CD11b and CD14 were relatively classic markers of differentiation in leukaemia. The results showed that E2F4 shRNA knockdown promoted the expression of CD11b and CD14 proteins in NB4 and THP‐1 cells (Figure 4C,G), whereas overexpression of E2F4 resulted in decreased expression of CD11b and CD14 (Figure 4D,H). Taken together, these data suggest that silencing E2F4 may contribute to leukaemia differentiation.

### 3.5 E2F4 can bind to EZH2 and regulate its expression in AML cells

**Figure 5:**
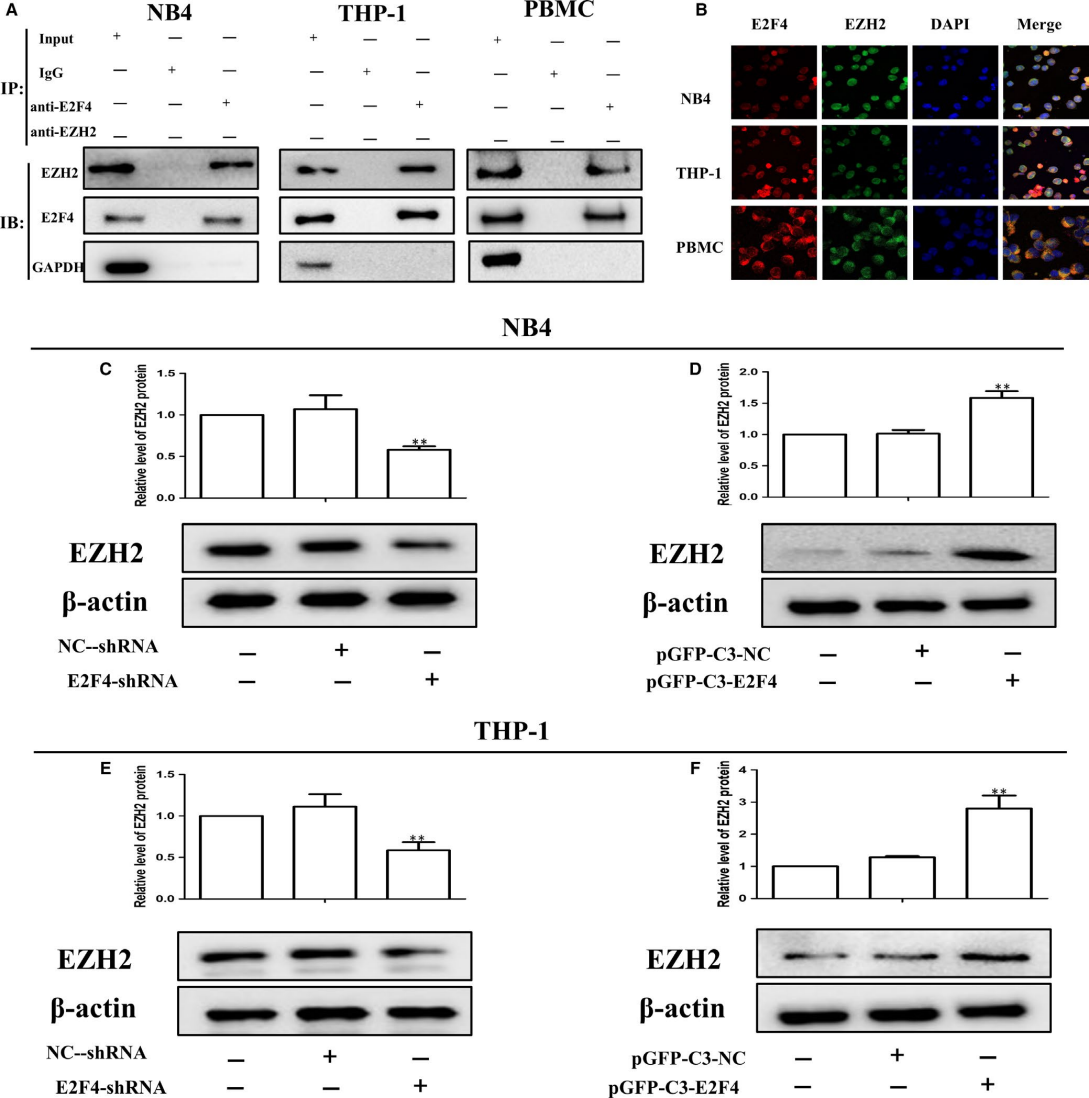
E2F4 can bind to EZH2 and regulate its expression in AML cells. A, The binding of E2F4 and EZH2 in the immunoprecipitation complex was validated by Western blotting. B, E2F4 and EZH2 colocalized to the cytoplasm. C, D, Western blot analysis of EZH2 protein levels in NB4 cells after E2F4 depletion or overexpression. E, F, Western blot analysis of EZH2 protein levels in THP‐1 cells after E2F4 depletion or overexpression. β‐Actin was used as the loading control. Data are presented as the mean ± SD of three independent experiments. *P < .05, **P < .01, compared with the NC group

The above findings demonstrate that E2F4 can regulate the proliferation and differentiation of leukaemia. We next performed Co‐IP and double immunofluorescent staining to analyse the binding of E2F4 and EZH2. The Co‐IP results showed significant binding of E2F4 and EZH2 in leukaemia cells and AML patients (Figure 5A). Furthermore, the immunofluorescence results showed that E2F4 and EZH2 localized to the cytoplasm (Figure 5B). The results also showed that E2F4 shRNA knockdown inhibited the expression of EZH2 protein in NB4 and THP‐1 cells (Figure 5C,5), whereas the overexpression of E2F4 promoted the expression of EZH2 (Figure 5D,5). In summary, E2F4 can interact with EZH2 to regulate its expression.

### 3.6 Overexpression of EZH2 reverses E2F4‐mediated inhibition of the MAPK signalling pathway

**Figure 6:**
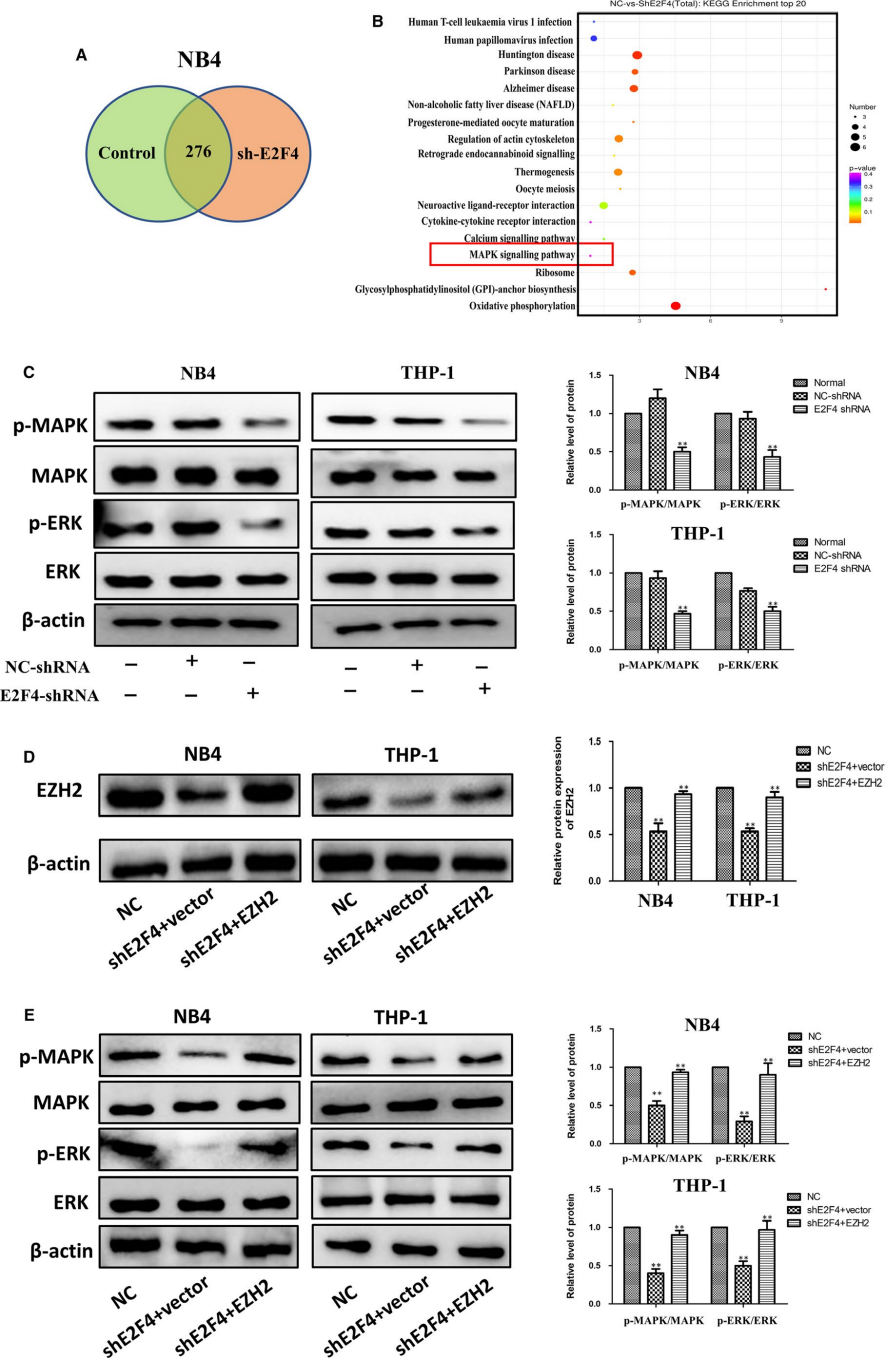
Overexpression of EZH2 reverses the inhibitory effect of E2F4 on the MAPK signalling pathway. A, Venn diagram showing the co‐altered genes of two independent shRNAs targeted to E2F4. B, KEGG analysis shows that E2F4 is involved in the regulation of MAPK signalling. C, Western blot analysis of ERK, p‐ERK, MAPK and p‐MAPK in NB4 and THP‐1 cells with transient silencing of E2F4 (shE2F4) or in NC cells without transfection. D, Relative protein expression of EZH2 was detected by Western blotting in NB4 and THP‐1 cells. E, ERK, p‐ERK, MAPK and p‐MAPK levels in NB4 and THP‐1 cells were measured by Western blotting. Data are presented as the mean ± SD of three independent experiments. *P < .05, **P < .01, compared with the NC group. ^#^ *P* < .05, ^# #^ *P* < .01, compared with the shE2F4 + vector group

To determine the mechanisms by which E2F4 induces AML cell proliferation and differentiation, we used an RNA microarray to examine the gene expression profiles of NB4 cells after transfection with either E2F4‐targeted shRNA or a negative control shRNA. We found 276 co‐altered genes for both shRNAs targeting E2F4 (Figure 6A). Subsequent gene ontology analyses indicated that the genes regulated by E2F4 were enriched for the regulation of the MAPK signalling pathway (Figure 6B). The MAPK signalling pathway is a classic cancer‐associated signalling pathway involved in the proliferation and differentiation of tumour cells.^18^, ^19^ The MAPK signalling pathway is also studied in AML.^20^, ^21^ To determine the specific mechanism of E2F4‐induced tumour cell proliferation and differentiation, we further examined the protein levels of MAPK, p‐MAPK, ERK and p‐ERK when E2F4 expression levels were manipulated in AML cells. Knockdown of E2F4 by shRNA significantly decreased the protein levels of p‐MAPK and p‐ERK compared with those in NB4 and THP‐1 cells transduced with NC‐shRNA (Figure 6C). To verify the regulatory relationship between E2F4 and EZH2, EZH2 was overexpressed in cell lines with stable E2F4 knockdown. The results showed that overexpression of EZH2 significantly restored the expression of EZH2 that was lost due to the silencing of E2F4 (Figure 6D). As expected, EZH2 overexpression also reversed the inhibition of MAPK signalling by E2F4 silencing in AML cell lines (Figure 6E). Taken together, these results indicate that E2F4 functions as a tumour suppressor in AML via inhibition of the MAPK signalling pathway by binding to EZH2.

### 3.7 Effects of E2F4 on tumour growth in vivo

**Figure 7:**
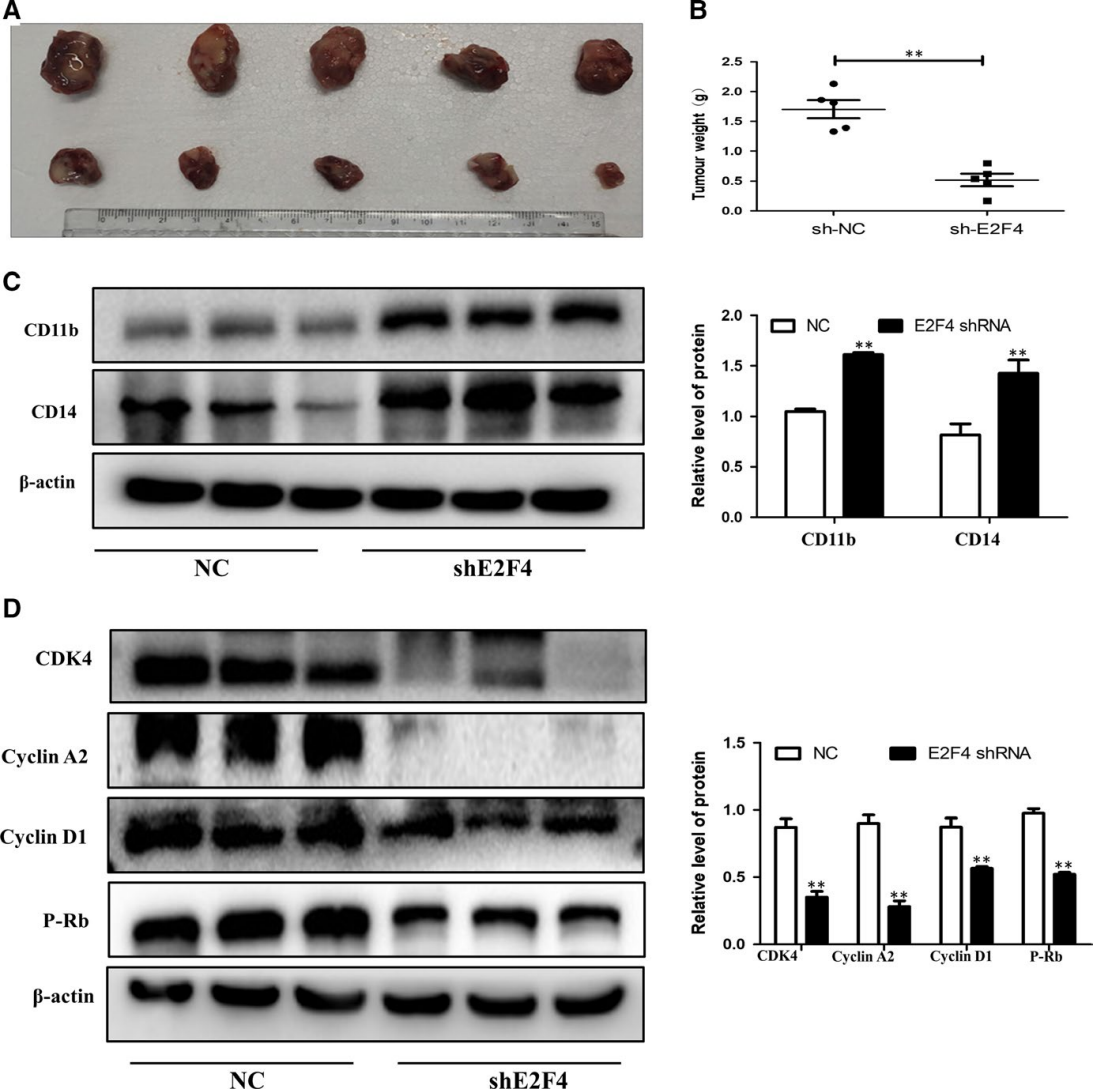
Effects of E2F4 on tumour growth in vivo. A‐C, Tumour images and weights at experimental end‐points in NC and shE2F4 NB4 xenografts (n = 5 for each group). D, Western blot analysis of CD11b and CD14 in tumour tissues of NC and shE2F4 groups. E, Western blot analysis of cyclin D1, cyclin A2, P‐Rb and CDK4 in tumour tissues of NC and shE2F4 groups. β‐Actin was used as an internal control. Bar graphs (mean ± SD) and representative images are shown. *P < .05, **P < .01, compared with the NC group

To verify the effect of the transcription factor E2F4 on tumour formation in vivo, we first established the stable cell line NB4, with E2F4 shRNA knockdown or with control shRNA as a negative control. The previous results verified the efficiency of silencing. Then, we injected shE2F4 or control NB4 cells subcutaneously into NCG nude mice to establish a tumour‐bearing mouse model. Tumour volume was measured every two days after tumour volume exceeded 100 cm^3^, and after 3 weeks, the mice were killed, and tumours were removed. The photographs and tumour volumes measured showed that cell growth was much slower in E2F4‐silenced cell tumours than in control cell tumours (Figure 7A). In addition, the weight of these tumours was much lower than that of the control tumours (Figure 7B). Western blotting showed that E2F4 knockdown promoted the expression of CD11b and CD14 and inhibited the expression of cyclin D1, cyclin A2, P‐Rb and CDK4 compared with the expression in control tumours (Figure 7C,7). Together, these data indicate that, as in the in vitro experiments, E2F4 may affect the proliferation and differentiation of AML tumours in vivo.

## 4 DISCUSSION

AML is a malignant haematological disease that results from different genetic and epigenetic abnormalities.^12^, ^22^, ^23^ In this study, we examined differentially expressed genes by microarray analysis and verified our results by analysing the data in public databases. In the present study, we analysed clinical data from the GEPIA database and found that increased E2F4 expression could reduce overall survival in patients with AML. This provides powerful clinical evidence that E2F4 may be related to the progression of AML. In our follow‐up study, we demonstrated the role of E2F4 in AML and examined the possible underlying mechanisms. Our results show that the expression of E2F4 in human AML patients and AML cell lines is higher than that in normal controls. These results indicate that E2F4 may play an oncogenic role in AML, affecting the occurrence and development of AML.

Leukaemia is caused by a block in differentiation. The treatment of this disease is mainly induced differentiation therapy.^24^, ^25^, ^26^, ^27^, ^28^, ^29^ The cell cycle is a series of events that leads to cell division and replication. Cell cycle progression may be limited by conditions that are not suitable for DNA replication, such as DNA damage, nutrient depletion and growth factor withdrawal.^30^, ^31^ The interaction between cyclins and cyclin‐dependent kinases (CDKs) regulates G1 phase and cell cycle progression as well as cell division,^32^, ^33^ which are important for driving cells past checkpoints.^34^ The proliferation and differentiation abilities of cells are the two most important features of malignant cell behaviour. Targeting the cell cycle, proliferation and differentiation is a potential approach to treating leukaemia. We found that knockdown of E2F4 expression inhibits the proliferation and induces the differentiation of AML. Furthermore, our results also indicate that overexpression of E2F4 promotes these behaviours in AML cells. Our next study also showed that knockdown of E2F4 induced cell cycle arrest in the G0/G1 phase. However, studies on the role of E2F4 on apoptosis and drug resistance in AML have not been carried out, and this role remains to be investigated.

The classic PI3K signalling pathway has an important effect on cell growth and proliferation through activation of cyclin D1.^35^ Our results show that E2F4 has a regulatory effect on cyclin D1 expression and AML proliferation. We also demonstrate that E2F4 targets the promoter of cyclin D1 and triggers its transcriptional activation. Previous studies have shown that activation of PI3K regulates E2F4 activation.^16^, ^36^ Therefore, E2F4 may be a downstream regulatory gene of the PI3K pathway; upon activation, E2F4 may subsequently alter the mRNA expression and protein levels of cyclin D1 to promote cell proliferation.

To explore the downstream regulatory pathway of E2F4, we used an RNA microarray to show that the MAPK signalling pathway may be downstream. Some reports indicate that MAPK dysregulation contributes to the development of colorectal, ovarian and breast cancers and AML.^20^, ^37^, ^38^, ^39^ Various cellular functions, such as apoptosis, proliferation, migration and invasion, are involved in MAPK‐dependent carcinogenesis.^40^, ^41^, ^42^ The MAPKs constitute a family of serine‐threonine kinases regulating the proliferation and differentiation of normal and malignant haematopoietic cells.^43^, ^44^ MAPK signalling and cell cycle regulatory pathways are dysregulated in nearly all melanomas, suggesting that cotargeting these pathways may be an attractive treatment strategy.^45^ These findings highlight the potential prognostic and therapeutic value of the MAPK signalling pathway in AML.

EZH2 is a histone methyltransferase component of PRC2. Both gain‐ and loss‐of‐function mutations of EZH2 have been involved in tumorigenesis.^13^, ^46^ Though down‐regulation and loss‐of‐function mutations suggest tumour‐suppressive activity of EZH2 in some cancers, there is evidence to demonstrate that EZH2 with gain‐of‐function mutations acts as an oncogene in numerous other cancers, such as prostate cancer and breast cancer (BC).^46^ Previously, we demonstrated that BRD4 facilitated EZH2 transcription through the recruitment of C‐MYC to the EZH2 promoter.^47^ E2Fs have also been reported to manipulate EZH2 transcription in several malignancies including BC.^48^ Rb/RB1 phosphorylation activated the expression of EZH2 through direct binding of E2F with the EZH2 promoter in BC and small cell lung cancer.^49^, ^50^ In this study, the Co‐IP assay and IF showed that E2F4 directly interacted with EZH2. These findings also indicated that down‐regulation of E2F4 inhibited AML cell proliferation and induced differentiation by inhibiting the expression of EZH2. Our results also showed that overexpression of EZH2 significantly restored the inhibition of MAPK signalling by E2F4 silencing in AML cell lines.

In the past few years, several potent inhibitors of EZH2 have been discovered. Roughly, they can be divided into S‐adenosyl‐L‐homocysteine (SAH) hydrolase inhibitors, such as 3‐deazaneplanocin A (DZNep); or S‐adenosyl‐L‐methionine (SAM) competitive inhibitors, such as GSK343.^51^, ^52^ In this study, we found that E2F4 and EZH2 are positively correlated. Silencing E2F4 can inhibit the expression of EZH2. Therefore, in the treatment of AML, in addition to the above EZH2 inhibitors, specific inhibitors can be developed for E2F4. In addition, E2F4 inhibitors may exert stronger inhibitory and therapeutic effects in AML than EZH2 inhibitors as EZH2 has a contrasting role in AML (during the induction phase, the presence of EZH2 improves survival, and during the maintenance phase, loss of EZH2 helps in survival). The treatment of AML is mainly divided into the induction therapy phase and the maintenance therapy phase. Given the different functions of EZH2 at different stages, it is worthwhile to explore whether E2F4 inhibitors can improve patient survival during the maintenance phase of AML.

Taken together, our results expanded our knowledge of the regulatory network between E2Fs and EZH2 in AML and confirmed that E2F4 functions as a tumour suppressor in AML via inhibition of the MAPK signalling pathway by binding to EZH2. Our study shows that E2F4 is a crucial modulator of human AML proliferation and differentiation via regulation of the MAPK signalling pathway in AML. EZH2 is a classic oncoprotein, and E2F4 can bind to EZH2 and regulate its expression, which may be another mechanism by which E2F4 can regulate AML. Overexpression of EZH2 significantly restored the inhibition of MAPK signalling that occurred with E2F4 silencing in AML cell lines. These data suggest that E2F4 may represent a new therapeutic target for AML therapy. In summary, E2F4 may become a target protein for leukaemia treatment.

## CONFLICT OF INTEREST

The authors have no conflicts of interest to disclose.

## Data Availability

The data used to support the findings of this study are available from the corresponding author upon request.
